# Amelioration of Inflammation in Rats with Experimentally Induced Asthma by *Spenceria ramalana* Trimen Polyphenols via the PI3K/Akt Signaling Pathway

**DOI:** 10.3390/ijms26010165

**Published:** 2024-12-28

**Authors:** Zhaobin Xia, Xing Zhao, Lu Wang, Lin Huang, Yanwen Yang, Xiangyu Yin, Luyu He, Yuebumo Aga, Ankaer Kahaer, Shiyu Yang, Lili Hao, Chaoxi Chen

**Affiliations:** 1 College of Animal Husbandry and Veterinary Medicine, Southwest Minzu University, Chengdu 610041, China; 210906012004@stu.swun.edu.cn (Z.X.); 210906012008@stu.swun.edu.cn (X.Z.); 80300002@swun.edu.cn (L.W.); huanglin200426@swun.edu.cn (L.H.); 80400016@swun.edu.cn (Y.Y.); 220906012010@stu.swun.edu.cn (X.Y.); 220952002052@stu.swun.edu.cn (L.H.); 220952002042@stu.swun.edu.cn (Y.A.); 220952002054@stu.swun.edu.cn (A.K.);; 2 Key Laboratory of Qinghai-Tibetan Plateau Animal Genetic Resource Reservation and Utilization, Southwest Minzu University, Chengdu 610041, China

**Keywords:** *Spenceria ramalana* Trimen, network pharmacology analysis, gut microbiota, fecal metabolomics, asthma, PI3K/Akt signaling pathway

## Abstract

Asthma is a chronic inflammatory respiratory disease that affects millions globally and poses a serious public health challenge. Current therapeutic strategies, including corticosteroids, are constrained by variable patient responses and adverse effects. In this study, a polyphenolic extract derived from the Tibetan medicinal plant *Spenceria ramalana* Trimen (SRT) was employed and shown to improve experimentally (ovalbumin + cigarette smoke, OVA + CS) induced asthma in rats. Initially, the potential therapeutic mechanism of the polyphenolic components in SRT on OVA + CS-induced asthma was predicated by network pharmacology analysis. Subsequently, in vivo experiments identified that SRT polyphenols exhibit significant anti-asthmatic activities, primarily mediated by lowering inflammatory cell counts such as the WBC (white blood cell), eosinophils, and neutrophils, decreasing the expression of inflammatory cytokines (IL-4, IL-5, IL-13, and TNF-α), alleviating lung histological damage (reduced inflammation, collagen deposition, and mucus secretion), and enhancing the epithelial barrier integrity (upregulation of ZO-1, occludin, and claudin-1). Additionally, SRT polyphenols downregulated the PI3K/Akt (Phosphoinositide 3-kinase/protein kinase B) signaling pathway, improved gut microbiota disruption, and regulated fecal metabolites (glucose-6-glutamate, PS (16:0/0:0), 8-aminocaprylic acid, galactonic acid, Ascr#10, 2,3,4,5,6,7-hexahydroxyheptanoic acid, phosphodimethylethanolamine, muramic acid, 9-oxohexadeca-10e-enoic acid, and sedoheptulose) in asthmatic rats. In conclusion, SRT polyphenols exerted multifaceted protective effects against OVA + CS-induced asthma in rats, highlighting their potential value in preventing asthma via the PI3K/Akt signaling pathway.

## 1. Introduction

Asthma, a chronic inflammatory respiratory disease, is characterized by airway inflammation and airflow limitation that are driven by allergens and environmental triggers and affects millions globally [1,2]. The pathophysiology of asthma involves complex processes, including airway epithelial cell damage, the release of inflammatory cytokines, mucus overproduction, and disruption of the gut microbiota [3,4]. The airway epithelial barrier, serving as the primary defense against harmful agents, maintains the stability of the intraepithelial environment [5]. Proteins such as zonula occludens-1 (ZO-1), occludin, and claudin-1 facilitate the formation of tight junctions (TJs) between epithelial cells, constituting a critical component of this barrier [6]. Numerous studies have shown that asthma is associated with compromised airway epithelial barrier function, characterized by altered expressions of ZO-1, occludin, and claudin-1, due to allergens, pathogens, and pollutants disrupting the TJs [7,8,9,10,11,12].

The pathogenesis of asthma is intricate, featuring eosinophilia and neutrophilia in blood or tissues as key phenotypes in mixed asthma. Asthma typically originates from disruption of the epithelial barrier, followed by continuous stimulation by pathogens. Various stimuli induce type 1 T helper cells (Th1) to release tumor necrosis factor-*α* (TNF-*α*), which drives neutrophil recruitment and increases airway smooth muscle contraction, ultimately facilitating asthma progression [13]. Simultaneously, type 2 T helper cells (Th2) secrete interleukin-4/5/13 (IL-4/5/13), leading to eosinophil accumulation, enhanced mucus production, and contraction of airway smooth muscle, exacerbating symptoms like coughing, wheezing, and dyspnea in bronchial asthma [14].

For patients with extrinsic asthma, the most effective approach involves promptly reducing allergen exposure through self-management and environmental control. Subsequently, pharmacological interventions are employed, with the primary objective of alleviating airway inflammation and preventing irreversible airway remodeling. Short-acting beta-agonists may be the first choice for quick asthma symptoms, and inhaled corticosteroids are usually chosen as medications. Dexamethasone, a traditional steroid with a longer half-life and powerful anti-inflammatory activities, is widely used for mild-to-moderate asthma exacerbations [15,16]. Currently, conventional therapies are hindered by variability in patient responses and the prevalence of adverse effects, which significantly limit their effectiveness in achieving comprehensive asthma management. Therefore, the development of innovative anti-asthma therapies is urgently needed. Given the complexity of asthma pathogenesis, targeting multiple molecular defects with a single therapeutic agent may be more effective than addressing a single pathological defect [17]. Natural products have garnered considerable attention in asthma treatment for their broad anti-inflammatory and antioxidant properties. Traditional medicine has acknowledged the therapeutic potential of plant extracts in asthma management for over 5000 years. In ancient China, the use of *Ephedra sinica* in decoctions was a customary practice, renowned for its efficacy in alleviating asthma symptoms. Building on this historical knowledge, modern research has demonstrated that numerous plant extracts and isolated compounds exhibit potent anti-asthmatic properties, including those derived from *Garcinia mangostana*, *Euphorbia hirta*, and *Paeonia emodi* Royle, among others [18]. An example is the natural compound nujiangexanthone A (N7), derived from *Garcinia nujiangensis*, which treats asthma by targeting Syk-dependent pathways and downstream molecules such as ERK and JNK, inhibiting mast cell degranulation and decreasing the release of cytokines (IL-4, IL-5, and IL-13) in an ovalbumin-induced asthma model. These findings highlight the therapeutic potential of natural compounds in asthma treatment [19].

*Spenceria ramalana* Trimen (SRT), the only species in the *Spenceria* genus within the Rosaceae family, is endemic to China, thriving in alpine grasslands at altitudes of 3000–5000 m in Sichuan and Yunnan Provinces and the Tibet Autonomous Region. It is recognized for its heat-clearing, detoxifying, and anti-inflammatory properties. Despite its traditional uses, few studies have focused on the pharmacological actions of SRT comprehensively, revealing a significant gap between traditional applications and scientific research. To date, only Lee et al. have explored the effects of isolated proanthocyanidins on vasodilation in the zebrafish hyaloid retina [20], while Chen et al. [21] and Tan et al. [22] have explored its potential in wound healing and ulcerative colitis treatment. To the authors’ knowledge, the anti-inflammatory effect and mechanism of the whole plant or active parts of SRT that are used to treat asthma treatment are unclear until now. In the present research, an OVA + CS-induced mixed asthma model in rats was established to assess the therapeutic efficacy of SRT polyphenols for asthma treatment, elucidating the underlying molecular mechanisms using network pharmacology analysis.

## 2. Results

### 2.1. Network Pharmacology Analysis of SRT Polyphenols for Asthma Treatment

**Figure 1:**
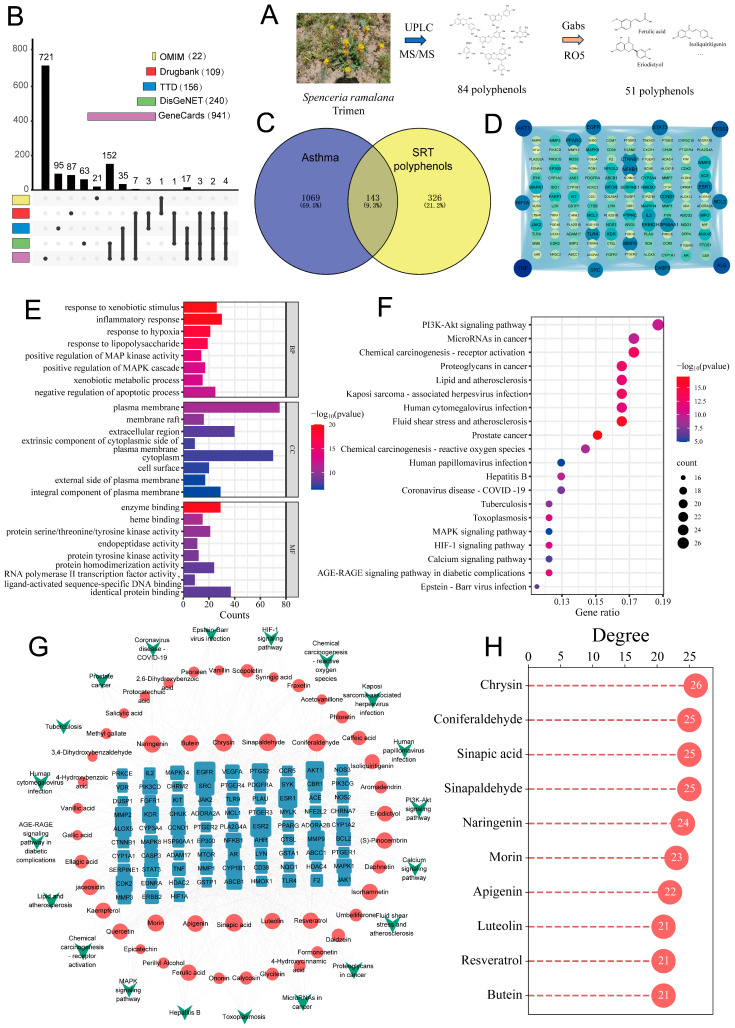
Network pharmacology analysis. (**A**) Flow chart for screening of potential active components. (**B**) Asthma-related targets from OMIM, Drugbank, TTD, DisGeNET, and GeneCards. (**C**) Venn diagram of 143 overlapping targets between SRT polyphenols and asthma. (**D**) PPI network of 143 overlapping targets. (**E**) GO function enrichment analysis. (**F**) KEGG enrichment analysis. (**G**) Component–target–pathway network (green nodes represent pathways, red nodes represent SRT polyphenols, and blue nodes represent targets). (**H**) Key components predicted for treatment of asthma.

Based on prior research, a total of 84 polyphenolic compounds were identified by Ultra-Performance Liquid Chromatography-Tandem Mass Spectrometry (UPLC-MS/MS) and systematically classified into 13 distinct categories according to their chemical structures, using comparative analysis with 130 polyphenol standards. These categories encompass flavonoids, benzoic acid and its derivatives, flavonols, phenylpropanoids, flavanones, coumarins and their derivatives, isoflavones, catechins and their derivatives, dihydrochalcones, stilbenes, anthocyanins, proanthocyanidins, and terpenoids [22]. Among the 84 polyphenolic compounds identified in SRT (Table S1), 51 potentially active components (Table S) were screened via “gastrointestinal absorption” (Gabs) and the “Rule of Five” (RO5) criteria (Figure 1A). Subsequently, SwissTargetPrediction identified a total of 469 targets associated with these polyphenols. Asthma-related target data were gathered from various databases, yielding 22 targets from OMIM, 109 from DrugBank, 156 from TTD, 240 from DisGeNET, and 941 from GeneCards (Figure 1B). Upon intersecting the asthma-related targets with those that were identified for the polyphenolic components, 143 overlapping targets were identified (Figure 1C). An interaction analysis among these overlapping targets suggested that the core targets of SRT polyphenols for asthma treatment encompass TNF, AKT1, ALB, PTGS2, STAT3, EGFR, CASP3, BCL2, HIF1A, and SRC (Figure 1D).

The GO analysis revealed that 911 biological function terms (*p* < 0.05), including 691 that were associated with biological processes (BPs), primarily involved a response to xenobiotic stimulus, inflammatory response, and response to hypoxia. The cellular component (CC) enrichment analysis yielded 81 entries, with the most prominent being the plasma membrane, membrane raft, and extracellular region. The molecular function (MF) enrichment analysis identified 139 terms, with significant associations in enzyme binding, heme binding, and protein serine/threonine/tyrosine kinase activity (Figure 1E). Additionally, the KEGG pathway enrichment analysis highlighted 152 significant signaling pathways (*p* < 0.05), with predominant pathways including the PI3K/Akt signaling pathway, chemical carcinogenesis-receptor activation, and microRNAs in cancer (Figure 1F). These SRT polyphenolic components, along with 20 significant signaling pathways and their respective targets, were integrated into the construction of a comprehensive component–target–pathway network. The resulting network consisted of 150 nodes, linked by 972 edges (Figure 1G). The ten key components, identified based on their degree values, were chrysin, coniferaldehyde, sinapic acid, sinapaldehyde, naringenin, morin, apigenin, luteolin, resveratrol, and butein (Figure 1H).

### 2.2. SRT Polyphenols Alleviated Inflammatory Responses

**Figure 2:**
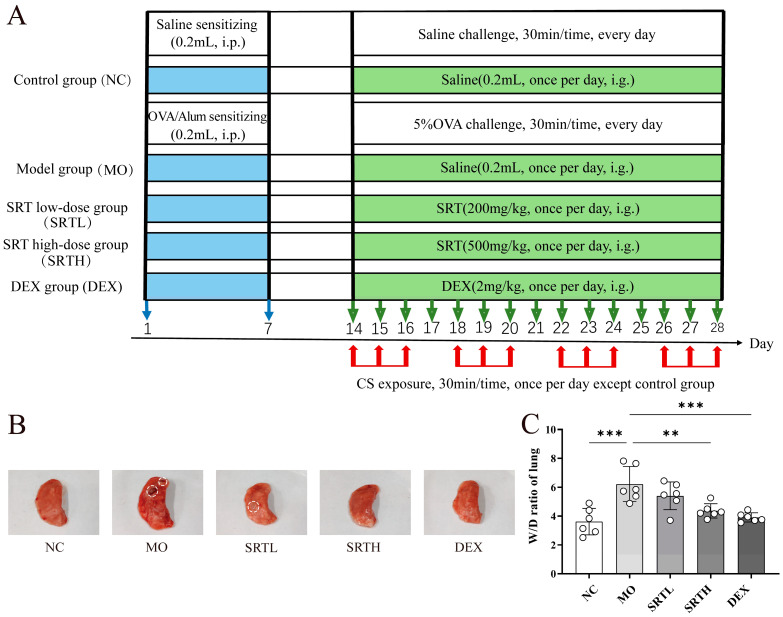
The establishment of an OVA + CS-induced asthma model. (**A**) Experimental design and dosing regimen. (**B**) Gross lesions in the lungs of asthmatic rats (white areas represent lesions and histological edema). (**C**) The W/D ratio of the lung. ** *p* < 0.01 and *** *p* < 0.001.

Prolonged exposure to OVA + CS (Figure 2A) induced more severe lung lesions in rats (Figure 2B), resulting in a substantially elevated wet/dry ratio of the lung (W/D) in the model (MO) group compared with the normal control (NC) group (*p* < 0.001). Treatment with SRT polyphenols high-dose (SRTH) significantly reduced the elevated W/D compared with the MO group (*p* < 0.001) (Figure 2C), suggesting that SRT polyphenols may alleviate histopathological lung lesions and edema in asthmatic rats [23].

**Figure 3:**
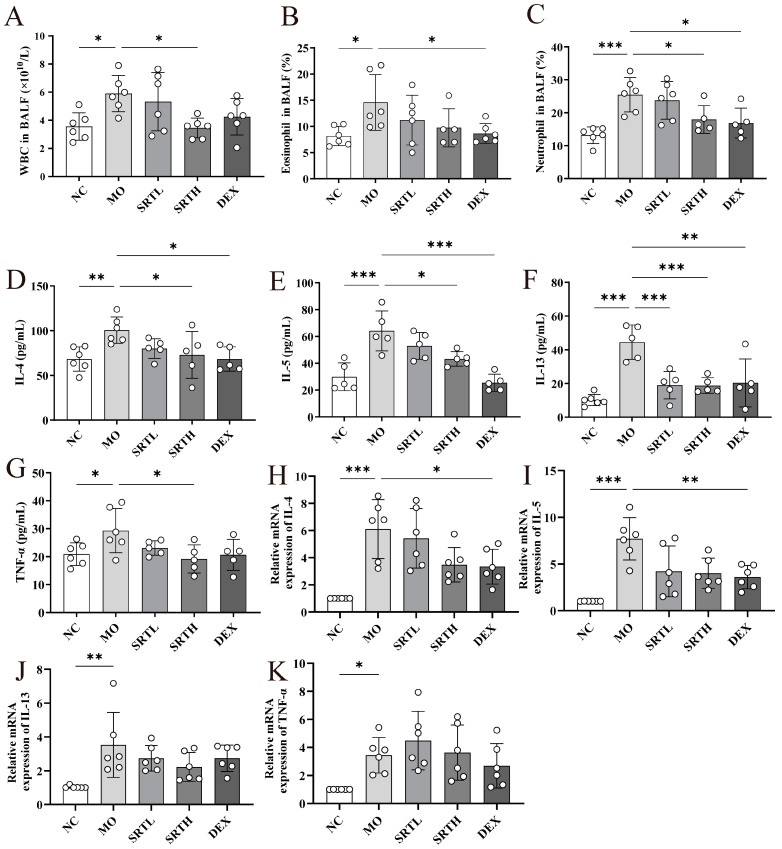
SRT polyphenols attenuated inflammatory responses in OVA + CS-induced asthmatic rats. (**A**–**C**) WBC count, percentages of eosinophils, and neutrophils in BALF. (**D**–**G**) ELISA-measured cytokine concentrations of IL-4, IL-5, IL-13, and TNF-α in serum. (**H**–**K**) Relative mRNA expression of *IL-4*, *IL-5*, *IL-13*, and *TNF-α* in lung tissues. Data are presented as mean ± SD. (n = 5–6). * *p* < 0.05, ** *p* < 0.01, and *** *p* < 0.001.

The total counts of WBCs, percentages of eosinophils, and neutrophils in BALF were markedly elevated in the MO group compared with the NC group (*p* < 0.05, *p* < 0.05, *p* < 0.001), and they were significantly reduced in both the SRTH and dexamethasone (DEX) groups relative to the MO group (*p* < 0.05, respectively) (Figure 3A–C). The ELISA results revealed significantly elevated concentrations of IL-4, IL-5, IL-13, and TNF-*α* in the MO group compared with the NC group (*p* < 0.01, *p* < 0.001, *p* < 0.001, *p* < 0.05). However, the inflammatory cytokine levels were significantly reduced in the SRTH group compared with the MO group (*p* < 0.05, *p* < 0.05, *p* < 0.001, *p* < 0.05) (Figure 3D–G). Moreover, exposure to OVA + CS significantly upregulated the mRNA expression levels of *IL-4*, *IL-5*, *IL-13*, and *TNF-α* compared with the NC group (Figure 3H–K). Treatment with SRT and DEX effectively suppressed this upregulated cytokine expression compared with the MO group. These findings indicate that SRT polyphenols may attenuate the inflammatory response in OVA + CS-induced asthmatic rats by inhibiting the cytokine expression levels.

### 2.3. SRT Polyphenols Repaired the Lung Epithelial Barrier

TJs, formed by epithelial cell adhesions, function as a physical barrier to maintain the stability of the intraepithelial environment. Disruption of this barrier elevates the lung permeability, facilitating exogenous pathogen invasion and retention, which subsequently trigger immune responses. Based on these mechanisms, the lung permeability in asthmatic rats was evaluated using Evans blue (EB) staining. Additionally, the expression levels of proteins associated with TJs, such as ZO-1, occludin, and claudin-1, were analyzed by Western blotting.

**Figure 4:**
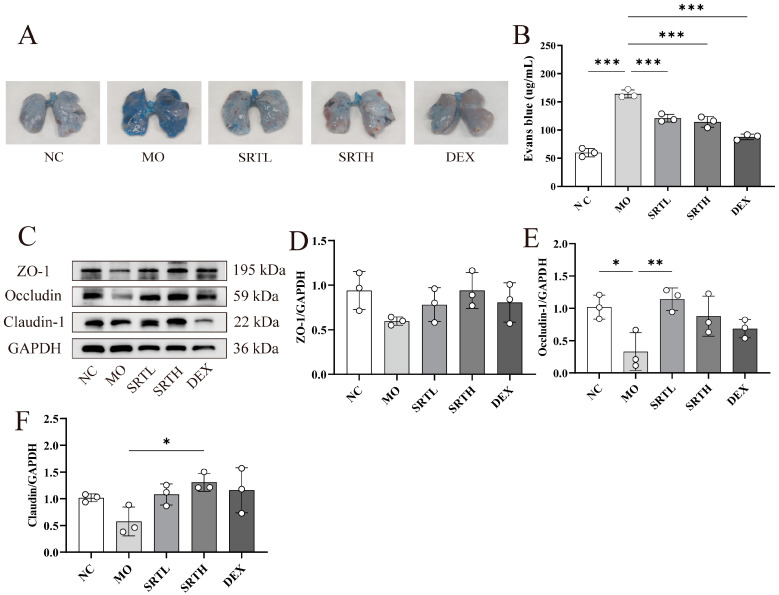
SRT polyphenols promoted lung epithelial barrier repair. (**A**) EB staining assay. (**B**) Quantification of dye in lung tissues. (**C**–**F**) Representative Western blot images and bar graphs showing relative expressions of ZO-1, occludin, and claudin-1. Data are presented as mean ± SD. (n = 3). * *p* < 0.05, ** *p* < 0.01, and *** *p* < 0.001.

As shown in Figure 4A,B, significant dye leakage was evident in the MO group, whereas treatments with SRT polyphenols low-dose (SRTL) and SRTH markedly reduced the leakage induced by OVA + CS (*p* < 0.001, respectively). The occludin expression was significantly decreased in the MO group compared with the NC group (*p* < 0.05) but was notably restored with SRTL treatment (*p* < 0.01). Similarly, SRTH treatment significantly enhanced the expression of claudin-1 compared with the MO group (*p* < 0.05). The ZO-1 expression was reduced following OVA + CS exposure but increased with SRT and DEX treatments; however, the changes were not statistically significant (Figure 4C–F).

### 2.4. Effects of SRT Polyphenols on Airway Remodeling

**Figure 5:**
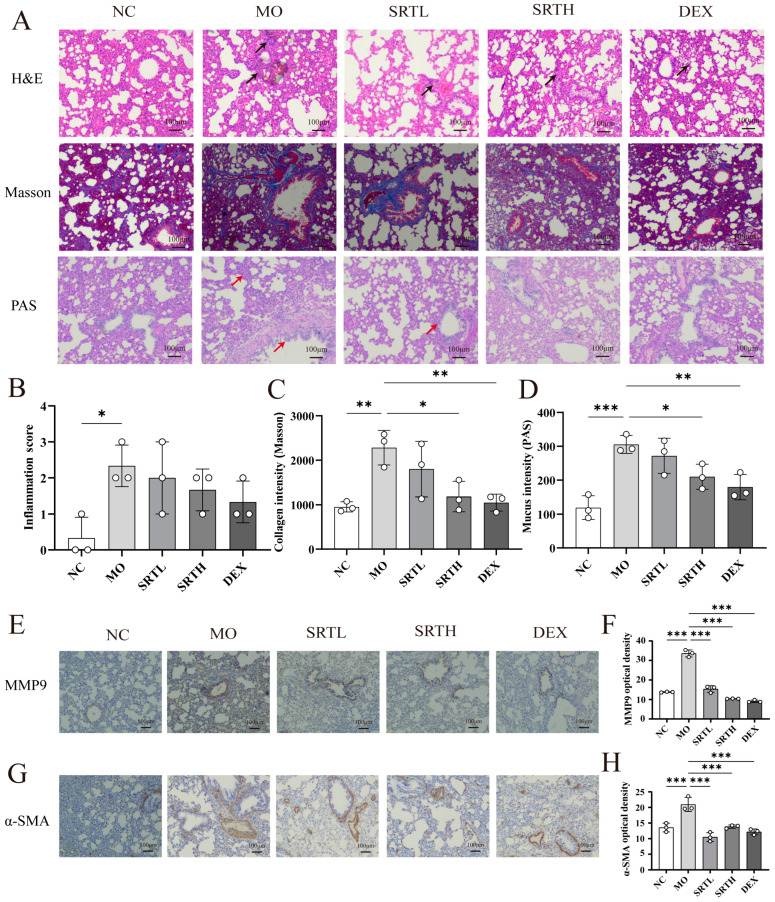
Effects of SRT polyphenols on airway remodeling. (**A**–**D**) H&E, Masson, and PAS staining of lung tissues with quantitative evaluation of inflammatory response, collagen deposition, and mucus secretion (black arrows in H&E staining indicate inflammatory cell infiltration; blue areas around airways in Masson staining represent collagen deposition; red arrows in PAS staining indicate mucus). (**E**–**H**) Immunohistochemical analysis for MMP9 and *α*-SMA with quantitative analysis. Data are presented as mean ± SD. (n = 3). * *p* < 0.05, ** *p* < 0.01, and *** *p* < 0.001.

The effects of SRT polyphenols on airway remodeling in asthmatic rats were evaluated using hematoxylin and eosin (H&E), Masson’s trichrome (Masson), periodic acid–Schiff (PAS) staining, and immunohistochemistry for MMP9 and α-SMA. The results indicated that the inflammatory score, collagen deposition, and mucus secretion were markedly elevated in the MO group compared with the NC group (*p* < 0.05, *p* < 0.01, *p* < 0.001). Following treatment with SRT or DEX, these markers decreased significantly (Figure 5A–D). The immunohistochemical analysis revealed a significant increase in the α-SMA and MMP9 expression levels in the MO group relative to the NC group (*p* < 0.001, respectively). As expected, these increases were significantly reversed by SRTH or DEX (*p* < 0.001, respectively) (Figure 5E–H).

### 2.5. SRT Polyphenols Modulated the PI3K/Akt Signaling Pathway

**Figure 6:**
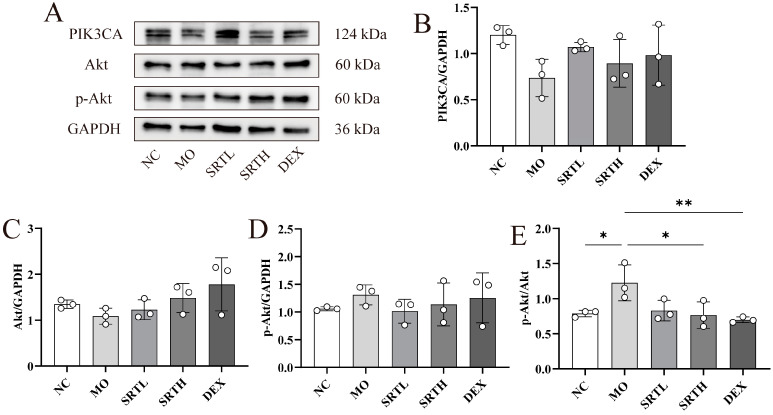
SRT polyphenols modulated the PI3K/Akt signaling pathways. (**A**–**E**) Representative Western blot images and bar graphs showing the relative expression levels of PIK3CA, Akt, and p-Akt. Data are presented as mean ± SD. (n = 3). * *p* < 0.05 and ** *p* < 0.01.

The PI3K/Akt signaling pathway is a canonical signaling axis and essential for regulating various biological processes in eukaryotic cells, including cell survival, growth, and cycle progression. Studies have demonstrated that allergic and inflammatory responses in asthma are mediated by the PI3K/Akt signaling pathway [24,25,26]. Network pharmacology analysis also suggests a potential role of this pathway in asthma treatment with SRT polyphenols. To validate the network pharmacology analysis and clarify potential mechanisms by which SRT alleviates OVA + CS-induced asthma, the protein expression levels in the PI3K/Akt signaling pathway were analyzed (Figure 6A–E). The expression of p-Akt/Akt was significantly elevated in the MO group compared with the NC group (*p* < 0.05), but markedly decreased following SRTH and DEX treatment (*p* < 0.05, *p* < 0.01) (Figure 6E). The expression level of PIK3CA proteins was reduced following OVA + CS exposure compared with the NC group, and the differences were not statistically significant (Figure 6B). However, neither exposure to OVA + CS nor treatment with SRT or DEX significantly affected Akt and p-Akt expression (Figure 6C,D).

### 2.6. SRT Polyphenols Regulated the Overall Structure of the Gut Microbiota

**Figure 7:**
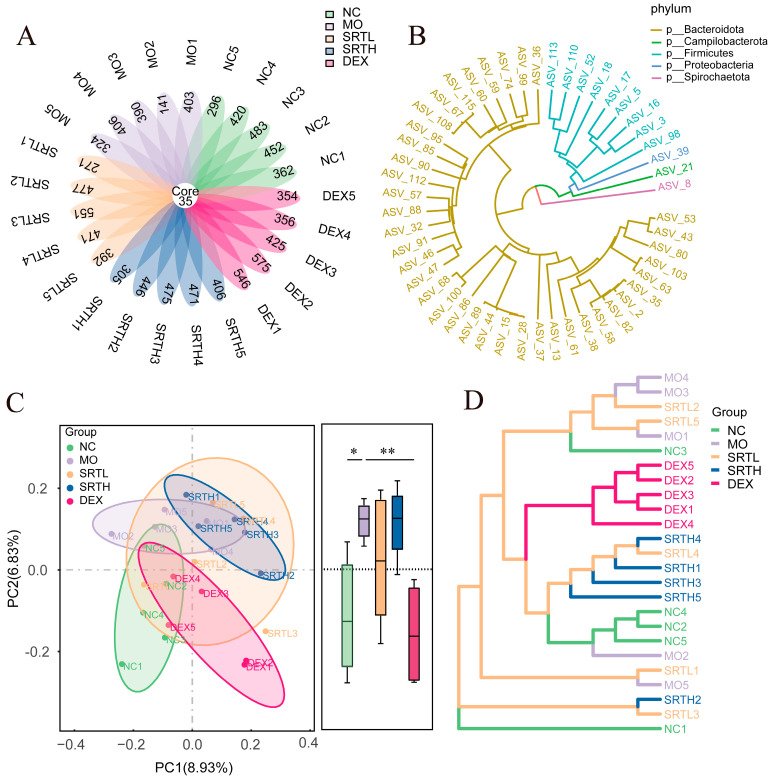
SRT polyphenols regulated the overall structure of the gut microbiota. (**A**) Petal plot of the ASV distribution. (**B**) Phylogenetic tree of the top 50 species. (**C**) Principal coordinates analysis (PCoA). (**D**) Hierarchical cluster analysis. Data are presented as mean ± SD. (n = 5). * *p* < 0.05 and ** *p* < 0.01.

Given the growing evidence supporting a “gut–lung axis” [27], we hypothesized that SRT polyphenols might regulate the gut microbiota structure while treating asthma in rats. Figure 7A presents the petal plots of the amplicon sequence variant (ASV), illustrating the number of identified and named species in each sample. Figure 7B represents the phylogenetic relationships among species. The principal co-ordinates analysis (PCoA) presented in Figure 7C revealed distinct microbial structural differences, especially along principal component 2 (PC2), with notable differences between the NC and MO groups (*p* < 0.05), which were substantially reversed following DEX treatment (*p* < 0.01). The microbial community structure of the NC group showed greater similarity to those of the SRTH and DEX groups. In contrast, the MO group displayed considerable dissimilarity and higher variability (Figure 7D). Additionally, we observed a decline in species diversity (observed_species, Chao1, and Shannon indices) in the MO group, which then increased following SRT or DEX treatment, and the differences were not statistically significant (Figure S1A–C). These findings suggest that SRT polyphenols may reverse the gut microbiota alterations induced by OVA + CS.

### 2.7. SRT Polyphenols Altered the Relative Abundance of the Gut Microbiota

**Figure 8:**
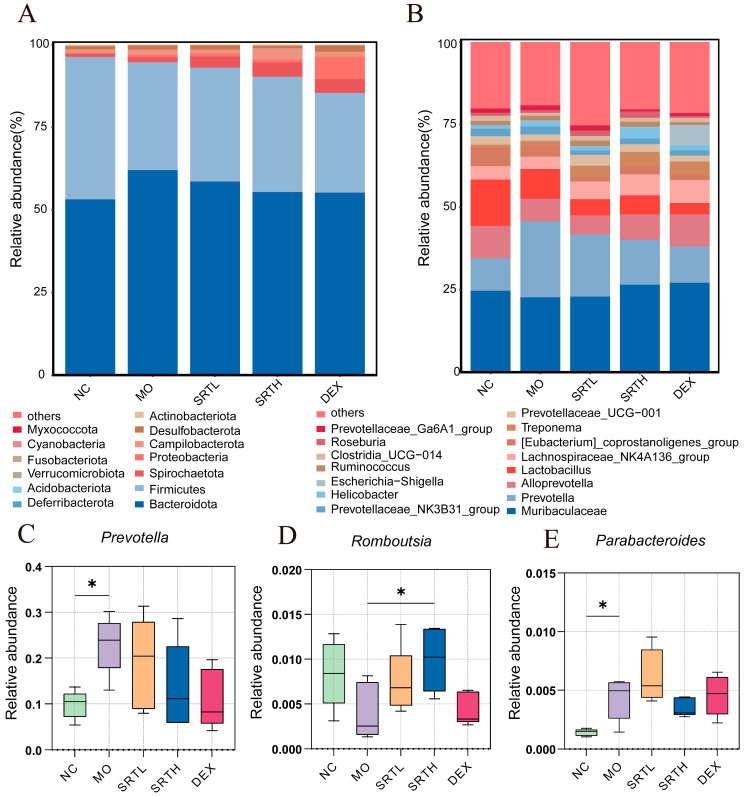
SRT polyphenols altered the relative abundance of the gut microbiota. Microbial community structure at the (**A**) phylum and **(B**) genus levels. (**C**–**E**) Representative differentially enriched species at the genus level: *Prevotella*, *Romboutsia,* and *Parabacteroides*. Data are presented as mean ± SD. (n = 5). * *p* < 0.05.

To elucidate the effects of SRT polyphenols on gut microbiota, we analyzed the relative abundances at both the phylum and genus levels (Figure 8A–E). Bacteroidetes and Firmicutes emerged as the predominant phyla, with OVA + CS exposure resulting in an increased relative abundance of Bacteroidetes and a decreased relative abundance of Firmicutes; these changes were partially reversed by SRT treatment (Figure 8A). The differentially enriched genera across all samples included *Muribaculaceae*, *Prevotella,* and *Alloprevotella*, which were predominant in the gut **(**Figure 8B). To visualize differences at the genus level more directly, Figure 8C–E were designed. *Prevotella* and *Parabacteroide*s significantly increased after OVA + CS exposure compared with the NC group (*p* < 0.05) and were restored to lower levels after SRTH treatment. Conversely, *Romboutsia* decreased in the MO group and increased significantly after SRTH treatment (*p* < 0.05).

### 2.8. LEfSe (Linear Discriminant Analysis Effect Size) Analysis

**Figure 9:**
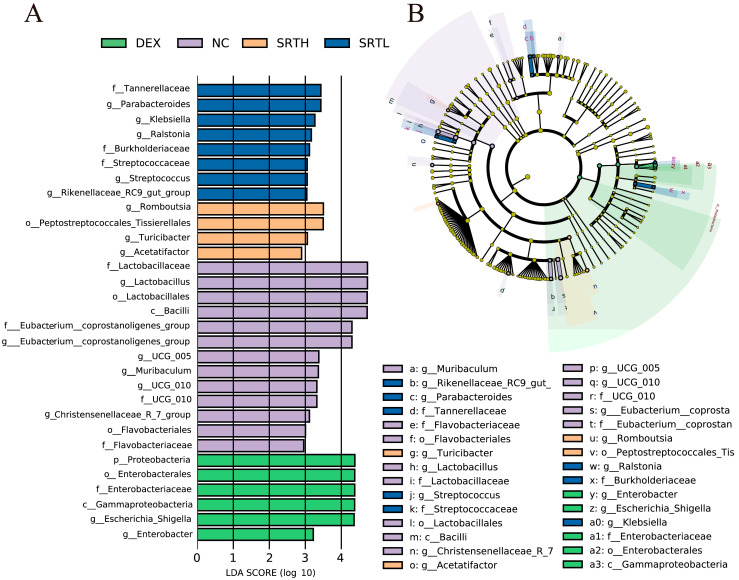
LEfSe analysis. (**A**) Differential species score chart and (**B**) differential species annotation branching diagram (n = 5).

Biomarker taxa were analyzed using LEfSe, which demonstrated a differential species score plot; bars of various colors represent different groups, and their lengths indicate effect sizes (Figure 9A). By integrating these specific highly enriched species into the LEfSe cladogram, we obtained Figure 9B. The results indicated that the NC group contained the greatest number of specific highly enriched species. However, surprisingly, the MO group did not possess any specific highly enriched species, which strongly supports our previous finding of decreased diversity in the MO group. At the genus level, the differentially enriched species included *Eubacterium_coprostanoligenes_group*, *Lactobacillus*, *UCG_0055*, *UCG_010*, *Muribaculum*, and *Christensenellaceae_R_7_group* in the NC group and *Romboutsia*, *Turicibacter*, and *Acetatifactor* in the SRTH group, along with *Escherichia_Shigella*, and *Enterobacter* in the DEX group. The LEFSe analysis revealed the biomarker profiles of the gut microbiota in each group, suggesting that SRT polyphenols could attenuate asthma by modulating the gut microbiota.

### 2.9. Modulation of SRT Polyphenols on the Fecal Metabolic Profiles

**Figure 10:**
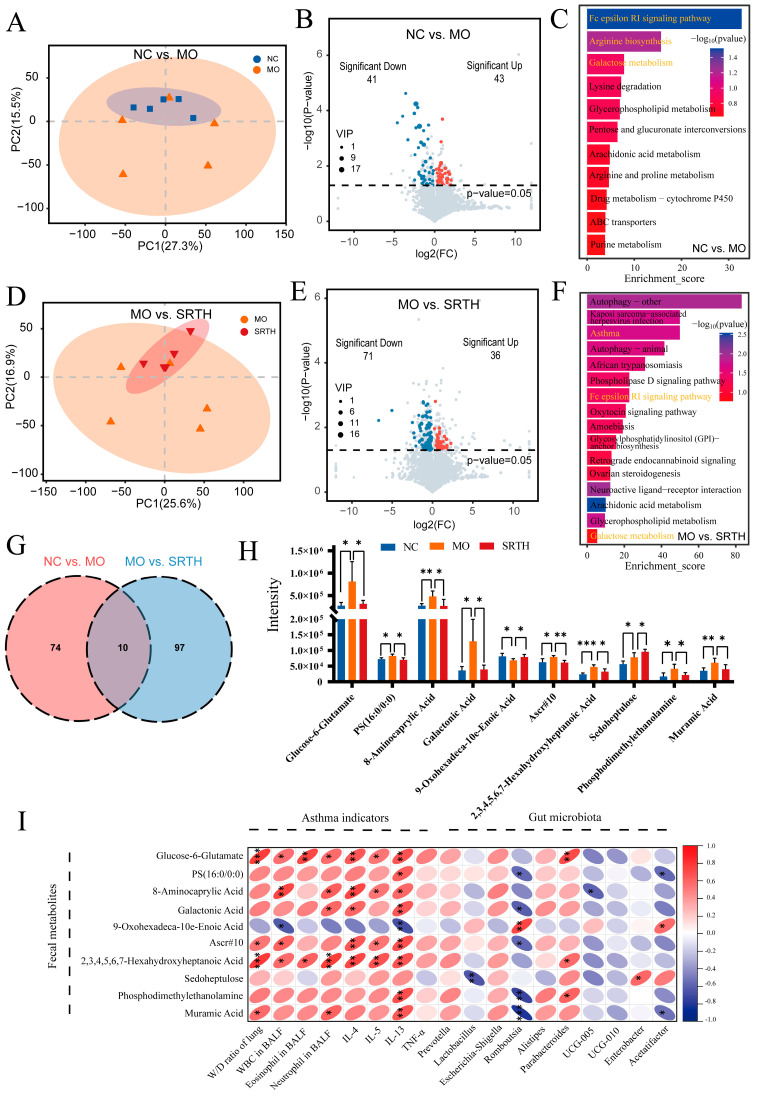
Fecal metabolome analysis. (**A**–**C**) PCA analysis, volcano plots of differentially expressed metabolites, and KEGG enrichment analysis for NC vs. MO. (**D**–**F**) PCA analysis, volcano plots of differentially expressed metabolites, and KEGG enrichment analysis for MO vs. SRTH. (**G**,**H**) Overlapping differentially expressed metabolites and expression levels between NC vs. MO and MO vs. SRTH. (**I**) Spearman’s correlation analysis of key differentially expressed metabolites with asthma indicators and gut microbiota. Data are presented as mean ± SD. (n = 5). * *p* < 0.05, ** *p* < 0.01, and *** *p* < 0.001.

An untargeted metabolomics analysis was conducted to investigate the effects of SRT polyphenols on fecal metabolites in asthmatic rats. The PCA demonstrated a distinct separation between the NC and MO groups, as well as between the MO and SRTH groups, with greater similarity being observed within the NC and SRTH groups and reduced similarity in the MO group (Figure 10A,D). Furthermore, differentially expressed metabolites were identified with VIP values > 1 and *p*-values < 0.05. A total of 84 significantly differentially expressed metabolites were identified between the NC and MO groups, with 41 downregulated and 43 upregulated. Similarly, a total of 107 metabolites were significantly differentially expressed between the MO and SRTH groups, with 71 downregulated and 36 upregulated (Figure 10B,E).

A KEGG enrichment analysis was performed to elucidate the functions of the differentially expressed metabolites (*p* < 0.05). Visualizing the pathways with the lowest *p*-values indicated that the three key pathways influenced by NC vs. MO differentially expressed metabolites were the Fc epsilon RI (FcεRI) signaling pathway, arginine biosynthesis, and galactose metabolism (Figure 10C). Consistent with our expectations, the differentially expressed metabolites in the MO vs. SRTH comparison were enriched in processes associated with asthma, galactose metabolism, and the FcεRI signaling pathway (Figure 10F). The results suggest that SRT polyphenols are involved in various asthma-related biological processes and signaling pathways, demonstrating the significant therapeutic potential for asthma treatment.

### 2.10. Identification and Correlation Analysis of Key Differentially Expressed Metabolites

Ten overlapping differentially expressed metabolites were identified in comparisons between NC vs. MO and MO vs. SRTH, potentially playing crucial roles in the treatment with SRT polyphenols (Figure 10G). Compared with the NC group, glucose-6-glutamate, PS (16:0/0:0), 8-aminocaprylic acid, galactonic acid, Ascr#10, 2,3,4,5,6,7-hexahydroxyheptanoic acid, phosphodimethylethanolamine, and muramic acid were significantly elevated in the MO group, with levels decreasing following SRTH treatment. Conversely, 9-oxohexadeca-10e-enoic acid decreased in the MO group but increased in the SRTH group, while sedoheptulose exhibited a progressive increase in both the MO and SRTH groups (Figure 10H).

Spearman’s correlation analysis was performed to assess the relationships between key differentially expressed metabolites, asthma indicators, and the top 10 genera of the gut microbiota (Figure 10I). Among the asthma indicators (W/D, WBC, eosinophil, neutrophils in BALF, IL-4, IL-5, IL-13, and TNF-α), positive correlations were observed with glucose-6-glutamate, PS (16:0/0:0), 8-ainocaprylic acid, galactonic acid, Ascr#10, 2,3,4,5,6,7-hexahydroxyheptanoic acid, sedoheptulose, phosphodimethylethanolamine, and muramic acid, while a negative correlation with 9-oxohexadeca-10e-enoic acid was observed. Conversely, all differentially expressed metabolites, except for 9-oxohexadeca-10e-enoic acid, demonstrated positive correlations with *Prevotella*, *Escherichia-Shigella*, *Alistipes,* and *Parabacteroides* while exhibiting negative correlations with *Romboutsia*, *UCG-005*, and *UCG-010*. Additionally, 9-oxohexadeca-10e-enoic acid was negatively correlated with *Prevotella*, *Escherichia-Shigella*, *Alistipes,* and *Parabacteroides* but positively with *Romboutsia* and *UCG-005*.

## 3. Discussion

Asthma is a chronic disease, characterized by variable and recurring airway inflammation, bronchial hyperresponsiveness, bronchoconstriction, vasodilatation, and airway edema. It is now understood as a heterogeneous, multifactorial disease that is influenced by genetic and environmental factors [28]. It is well established that Th1/Th2 imbalance is a critical factor in the pathogenesis of asthma. OVA, a prevalent allergen, is related to type 2 asthma, which is characterized by mucus secretion, tissue hyperplasia, and a significant elevation in eosinophil counts [29,30]. Initial exposure to OVA activates Th2 cells, increasing Th2 cytokines (including IL-4, IL-5, and IL-13) and triggering a cascade of asthmatic responses. IL-4 promotes Th2 cell differentiation, B-cell IgE isotype switching, and the upregulation of FcεRI on target cells, thereby enhancing inflammatory cytokine secretion; IL-5 primarily facilitates eosinophil activation and recruitment, leading to bronchial inflammation; and IL-13 is chiefly responsible for mucus production and collagen deposition, which contributes to airway thickening. These mechanisms underpin the primary symptoms of asthma, including breathlessness and persistent mucus secretion [14,31,32]. Chronic exposure to CS, a common inhalant irritant, recruits neutrophils and promotes epithelial cell proliferation, thereby inducing an inflammatory response in the airways driven by Th1 cells and TNF-α secretion, which differs from typical type 2 asthma [13,33,34,35].

SRT, as an essential natural resource and medicinal plant, has been used by Tibetans in Sichuan province; however, comprehensive pharmacological research is extremely lacking, leading to an imbalance between various folk applications and advanced developments. In this study, we focus on gut flora and fecal metabolites to explore the connections with asthma, shedding light on the link between the two organs and our understanding of the newly proposed concept of the “gut–lung axis”. Our findings indicate that the levels of IL-4/5/13, TNF-α, eosinophils, and neutrophils were significantly elevated in the mixed asthma model induced by OVA + CS and decreased following treatment with SRT polyphenols, particularly at higher doses, revealing that SRT polyphenols have anti-inflammatory pharmacological effects on asthma. We also found that fecal metabolites were closely related to asthma indicators and gut flora through correlation analysis, verifying the vital effect of the “gut–lung axis” on ameliorating OVA+ CS-induced asthma after SRT polyphenol treatment.

Given its crucial role in regulating airway inflammation, the inhibition of the PI3K/Akt signaling pathway through targeted molecules that modulate Th1/Th2 responses has been shown to mitigate the pathological changes associated with asthma and contribute significantly to airway protection [32]. Concurrently, the activation of the PI3K/Akt signaling pathway promotes bronchial smooth muscle contraction, resulting in airway narrowing and dyspnea [36]. In the current study, the network pharmacology analysis predicted that SRT polyphenols may exert therapeutic effects in asthma by modulating the inflammatory response via the PI3K/Akt signaling pathway, and the animal experiments verified that the pharmacological effects are mediated through the inhibition of the PI3K/Akt signaling pathway. In addition, the histopathological analysis demonstrated a mitigated inflammatory response, reduced collagen deposition, and decreased mucus secretion in lung tissues following SRT polyphenol treatment. Previous research has identified α-SMA as a critical marker of airway smooth muscle cells, with elevated levels indicating the presence of lung fibroblasts [37]. Elevated levels of MMP9, a member of the metalloproteinase family, typically promote collagen deposition in the airways, thereby enhancing fibrosis and exacerbating airway inflammation [38]. Our results also indicated that SRT polyphenols significantly inhibited the elevated expression of α-SMA and MMP9, underscoring their potential to effectively alleviate inflammatory responses, which is crucial for therapeutic efficacy in asthma treatment.

Another significant issue in asthma is the disruption of the pulmonary barrier [8,13]. This compromised barrier facilitates pathogen invasion and colonization, exacerbating the inflammatory response and establishing a vicious cycle. Therefore, restoring the homeostasis of the pulmonary epithelial barrier is essential for reinforcing the body’s first line of defense against exogenous substances. Our results demonstrated that the administration of SRT polyphenols improved indices of pulmonary leakage and enhanced the expression of ZO-1, occludin, and claudin-1, with more pronounced effects in the SRTH group that were comparable to those observed with DEX.

An increasing body of evidence supports gut–lung crosstalk, referred to as the “gut–lung axis”, wherein the microbiota plays a significant role [3,39,40]. Numerous studies have demonstrated that individuals with asthma exhibit gut dysbiosis and reduced microbial diversity, indicating a strong correlation between gut microbiota and asthma [41,42,43,44]. However, additional evidence is required to ascertain whether pharmacological interventions for preventing and treating asthma can ameliorate gut dysbiosis. Alterations in microbiota diversity and relative abundance were observed in the MO and SRT polyphenol treatment groups; SRT treatment increased the abundance of *Romboutsia* while decreasing the abundance of *Prevotella* and *Parabacteroides* compared with the MO group. The increased abundance of *Parabacteroides* in the asthma model aligns with findings from Wu et al. [45], where *Parabacteroides* has been identified as a potentially pro-inflammatory bacterium that is more prevalent in individuals with asthma or other inflammatory diseases [46]. Conversely, our observations of a decrease in *Romboutsia* within the asthma model group contradict those reported by Wu et al. [45]. However, they are consistent with findings from Hu et al. and Liu et al. [47,48]. *Prevotella* is known for its pro-inflammatory properties, and a high relative abundance of *Prevotella* in the airway microbiota during early infancy is associated with an elevated risk of developing asthma later in life [49,50,51,52]. Our experiments indicated an increased relative abundance of *Prevotella* in the MO group, corroborating the results of Fazlollahi et al. [53]. The results indicate that in OVA + CS-induced asthmatic rats, the gut microbiota exhibits structural dysbiosis and reduced diversity, along with an elevation in *Prevotella* and *Parabacteroides* associated with inflammation. This dysbiosis may be ameliorated through treatment with SRT polyphenols.

The fecal metabolite analysis revealed that differentially expressed metabolites in the NC vs. MO group were enriched in the FcεRI signaling pathway, arginine biosynthesis, and galactose metabolism, indicating the development of asthma in the MO group of rats. FcεRI is pivotal in allergic and inflammatory responses. Mast cells, central to the allergic response, release cytokines and leukotrienes by activating the FcεRI receptor on their surface, triggering allergic and inflammatory reactions [54,55]. Arginase plays a key role in the competing synthesis of nitric oxide, with increased arginase activity enhancing nitric oxide production in the bronchioles, thereby intensifying the airway response and facilitating the progression of asthma [56]. Zhou et al. found that serum metabolites in asthma patients were primarily enriched in arginine biosynthesis [57]. Our study observed a similar enrichment in the gut metabolites of asthmatic rats, highlighting the critical role of arginine in asthma progression and the link between gut metabolites and asthma via blood circulation, reinforcing the significance of the “gut–lung axis”. Previous research indicated that differential metabolites in the serum or BALF of asthma patients were predominantly found in the galactose metabolic pathway [58,59]. Our study similarly found that differential metabolites in the guts of asthmatic rats were enriched in the galactose metabolic pathway. Additionally, galacturonic acid, a crucial metabolite linked to oxidative stress [60], exhibited elevated expression levels in the MO group and was significantly reduced following SRT polyphenol treatment. Differentially expressed metabolites from MO vs. SRTH were enriched in the FcεRI signaling pathway, arginine biosynthesis, and asthma bioprocesses, indicating the therapeutic potential of SRT polyphenols for asthma management.

Furthermore, a total of 10 overlapping differentially expressed metabolites were identified in the comparisons of NC vs. MO and MO vs. SRTH, including glucose-6-glutamate, PS (16:0/0:0), 8-aminocaprylic acid, galactonic acid, 9-oxohexadeca-10e-enoic acid, Ascr#10, 2,3,4,5,6,7-hexahydroxyheptanoic acid, sedoheptulose, phosphodimethylethanolamine, and muramic acid. Previous research has reported that elevated levels of galacturonic acid are associated with oxidative stress [60]. It is hypothesized that bacterial debris accumulates in tissues and acts as an inflammatory trigger in human diseases [61]. Muramic acid, a major component of bacterial debris, has been detected in the cerebrospinal fluid of patients with pneumococcal meningitis, and higher concentrations of muramic acid are linked with increased asthma severity [62,63]. Finally, the correlation analysis in this study highlighted significant associations between key differentially expressed fecal metabolites, gut microbiota, and asthma indicators in the OVA + CS-induced asthma model. These results emphasize the pivotal role of the “gut–lung axis” in asthma pathogenesis and demonstrate the ability of SRT polyphenols to modulate these interactions, restoring the microbial balance and alleviating inflammation. This comprehensive analysis underscores the therapeutic potential of targeting gut microbiota and metabolites for asthma management and advocates for further investigations into the underlying mechanisms of natural polyphenols.

**Figure 11:**
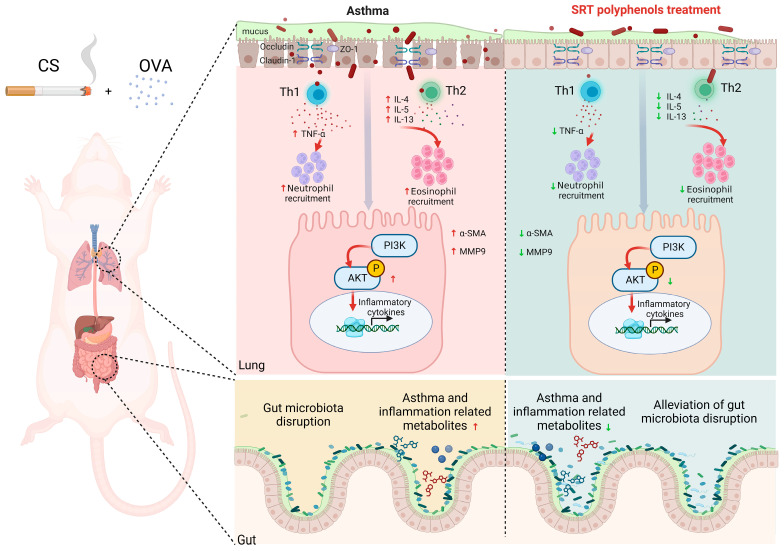
The underlying mechanism of the protective effect of SRT polyphenols against OVA + CS-induced asthma.

The potential protective mechanism of SRT polyphenols against OVA + CS-induced asthma is described in Figure 11. There are still limitations of this study; for example, inhibitors were not employed to demonstrate the downregulated effect of SRT polyphenols in the PI3K/Akt signaling pathway. Furthermore, a dose–effect relationship analysis of SRT polyphenols will be carried out against OVA + CS-induced asthma in our ongoing study.

## 4. Materials and Methods

### 4.1. Chemicals and Reagents

Evans blue (EB, cat: S19046), dexamethasone (DEX, cat: S17003), and leukocyte diluent solution (cat: R20340) were purchased from Shanghai Yuanye Biotechnology Co., Ltd. (Shanghai, China). Ovalbumin (OVA, cat: A18695) was obtained from Xiya Reagent^®^ (Linshu, China). Cigarettes (PRIDE) were provided by China Tobacco Sichuan Industrial Co., Ltd. (Chengdu, China), and Wright-Giemsa staining solution was purchased from BKMAN Biotechnology Co., Ltd. (Changde, China). Enzyme-linked immunosorbent assay (ELISA) kits for interleukin-4 (IL-4, cat: H005-1-1), interleukin-5 (IL-5, cat: H006-1-1), interleukin-13 (IL-13, cat: H011-1-1), and tumor necrosis factor *α* (TNF-*α*, cat: H052-1-1) were purchased from Nanjing Jiancheng Bioengineering Institute (Nanjing, China). Primary antibodies (ZO-1, cat: AF8394; occludin, cat: AF7644; claudin-1, cat: AF6504), phospholipase C regulates activation of class 1 (PIK3CA, cat: AF7749), protein kinase B (Akt, cat: AA326-1), phosphorylated Akt (p-Akt, cat: AA329-1), glyceraldehyde 3-phosphate dehydrogenase (GAPDH, cat: AF1186), and goat anti-rabbit IgG (H + L) secondary antibody (cat: A0208) were purchased from Beyotime Biotechnology Co., Ltd. (Shanghai, China). Additionally, primary antibodies against alpha-smooth muscle actin (*α*-SMA, cat: GB111364-100) and matrix metallopeptidase 9 (MMP9, cat: GB11132-100) were obtained from Service Biotechnology Co., Ltd. (Wuhan, China).

### 4.2. Plant Material and Experimental Animals

In October 2019, the whole plant of SRT was collected from Danqing Yakwang Shenshan (32°11′27′′ N, 100°25′48′′ E, approximately 3900 m above sea level), located in Seda County, Ganzi Prefecture, Sichuan Province, and was authenticated by associated professor Chaoxi Chen of Southwest Minzu University, Chengdu, Sichuan, China. The voucher specimens were deposited in the Laboratory of Veterinary Pharmacology and Toxicology at Southwest Minzu University.

Adult male specific-pathogen-free (SPF)-grade Sprague-Dawley rats (180–200 g) were acquired from Chengdu Dossy Experimental Animals Co. Ltd. [The License Numbers of Laboratory Animals: SCXK(Sichuan)2020-030]. The animals underwent a 7-day acclimatization period with unrestricted access to clean drinking water and standard pelleted feed throughout the experimental period. All experimental procedures were approved by the Institutional Animal Care and Use Committee (IACUC) of Southwest Minzu University.

### 4.3. Identification of Major Components of SRT Polyphenols

The identification and characterization of SRT polyphenols were described in our prior publication [22]. In brief, two kilograms of dried SRT were finely pulverized and macerated with 50% ethanol (1:5, *v*/*v*) for 33 min under ultrasound conditions. The extraction procedure was repeated three times, and the filtered extract was concentrated at 55 °C under reduced pressure using a rotary evaporator. The identification of polyphenolic components was performed using a UPLC-MS/MS system. Finally, the SRT polyphenols were freeze-dried and used for subsequent animal experiments.

### 4.4. Network Pharmacology Analysis of Active Components of SRT Polyphenols for Asthma Treatment

The absorption of chemical components into the bloodstream and their therapeutic efficacy are influenced by their gastrointestinal absorption characteristics and drug-like properties, as assessed by “gastrointestinal absorption” (Gabs) and the “Rule of Five” (RO5) [64]. The SMILES notations of SRT polyphenols, identified via UPLC-MS/MS, were retrieved from PubChem (https://pubchem.ncbi.nlm.nih.gov/, accessed on 4 January 2023), and their ADME properties were evaluated using SwissADME (http://www.swissadme.ch/, accessed on 4 January 2023) to identify potential active components with high Gabs that meet the RO5 criteria.

Targets for potential active components were predicted using the SwissTargetPrediction database (http://swisstargetprediction.ch/, accessed on 4 January 2023), and asthma-related targets were obtained from the OMIM (https://omim.org/, accessed on 4 January 2023), Drugbank (https://go.drugbank.com/, accessed on 4 January 2023), TTD (http://db.idrblab.net/ttd/, accessed on 4 January 2023 ), DisGeNET (https://www.disgenet.org/, accessed on 4 January 2023), and GeneCards (https://www.genecards.org/, accessed on 4 January 2023) databases. Overlapping targets between SRT polyphenolic active components and asthma were identified using Venny2.1.0 (https://bioinfogp.cnb.csic.es/tools/venny/, accessed on 4 January 2023) and further analyzed in STRING11.5 (https://www.string-db.org/, accessed on 4 January 2023) to construct a protein–protein interaction (PPI) network and screened for key targets using Cytoscape 3.8.2. These overlapping targets were then analyzed using DAVID (https://david.ncifcrf.gov/, accessed on 4 January 2023) for gene ontology (GO) and Kyoto Encyclopedia of Genes and Genomes (KEGG) pathway enrichment analysis. A “component–target–pathway” network was constructed using Cytoscape 3.8.2, with key components identified based on their degree values.

### 4.5. Pharmacological Effects of SRT Polyphenols on OVA + CS-Induced Asthma in Rats

#### 4.5.1. OVA + CS-Induced Asthma Model

The OVA + CS-induced asthma model was established according to references [65,66] with minor modifications. Initially, forty-five rats were randomly assigned to five groups: normal control (NC), model (MO), SRT polyphenols low-dose (SRTL), SRT polyphenols high-dose (SRTH), and dexamethasone (DEX). On days 0 and 7, the NC group received intraperitoneal injections of 0.2 mL saline, while the MO, SRTL, SRTH, and DEX groups received 0.2 mL OVA/Alum mixture (20 μg OVA + 1 mg aluminum hydroxide + 200 μL saline). Beginning on day 14, all groups except the NC were exposed daily to nebulized 5% OVA. CS exposure was implemented in three-day cycles with a one-day break between cycles, continuing until day 28. Additionally, the NC and MO groups received a daily gavage of 0.2 mL saline. In contrast, the DEX, SRTL, and SRTH groups were administered daily gavages of 2 mg/kg DEX, 200 mg/kg SRT, and 500 mg/kg SRT, respectively. These dosages were determined based on prior antitussive and anti-asthmatic studies. On day 28, all rats were euthanized using chloral hydrate, and samples of serum, lung tissues, bronchoalveolar lavage fluid (BALF), and feces were collected for analysis.

#### 4.5.2. Pulmonary Permeability and W/D Ratio

The Evans blue (EB) assay was performed as follows [67]: Four hours after the last administration, three rats per group received an intravenous EB injection (50 mg/kg) via the tail vein. An hour post-injection, the rats were anesthetized and perfused with saline through the right ventricle for 5 min. The lungs were then homogenized in 1 mL of phosphate-buffer solution (PBS) and extracted in 2 mL of formamide at 60 °C for 18 h. The EB concentration was determined using a standard curve at OD_640_ and OD_720_.

The unirrigated left lung was weighed to obtain its wet weight (W) and then oven-dried at 60 °C for 72 h to a constant weight, after which the dry weight (D) was recorded to calculate the W/D ratio.

#### 4.5.3. Inflammatory Cell Counts in BALF

After the rats were euthanized, the chest cavity was carefully opened to expose the trachea. A small incision was made, and a sterile gavage needle was inserted into the right bronchus base before the trachea was secured with sutures. The left bronchus was isolated and ligated with sutures to prevent contamination. A syringe containing 2 mL of saline was attached to the gavage needle and gently injected into the right lung. After a 30 s retention, the saline was withdrawn to collect BALF. This procedure was repeated three times, yielding approximately 3 mL of BALF per rat.

As previously described [68], the BALF samples were centrifuged at 3000 rpm for 10 min, and the pellet was resuspended in 200 μL of PBS. A fraction of this suspension was mixed with a leukocyte diluent and applied to a cell counting plate, where the total white blood cell (WBC) count was determined microscopically (Olympus Corporation, Tokyo, Japan). The remaining resuspended samples were centrifuged (3000 rpm, 10 min), smeared, fixed, and stained with Wright–Giemsa stain to assess the percentages of eosinophils and neutrophils.

#### 4.5.4. Cytokine Concentration in Serum

The cytokine concentrations of IL-4, IL-5, IL-13, and TNF-α were measured according to the manufacturer’s instructions: the test serum was added to the sample plate and incubated at 37 °C, then washed, exposed to enzyme reagent for 30 min, and rewashed. Following the development of color, the reaction was terminated, and the absorbance was measured using a SpectraMax iD3 (Molecular Devices Corporation, San Jose, CA, USA). The cytokine concentrations were then calculated based on the standard curves.

#### 4.5.5. Gene Expression of Cytokines in Lung Tissues

Quantitative reverse transcription polymerase chain reaction (RT-qPCR) was performed to measure the cytokine gene expression in lung tissues. RNA was extracted from lung tissue using a TRIzol kit (Service Biotechnology Co., Ltd., Wuhan, China) and reverse-transcribed into cDNA using a reverse transcriptase kit (Service Biotechnology Co., Ltd., Wuhan, China). The cDNA, primers (Table S3), and SYBR Green qPCR Master Mix (Service Biotechnology Co., Ltd., Wuhan, China) were used for PCR amplification on a real-time PCR system (Bio-Rad Laboratories, Inc., Hercules, CA, USA). The relative expression levels of inflammatory cytokines were analyzed using the 2^−ΔΔCt^ method, with *β*-actin serving as the reference gene.

#### 4.5.6. Western Blotting

Total proteins were extracted from lung tissues following the RIPA protocol (Beyotime Biotechnology Co., Ltd., Shanghai, China), and concentrations were determined by a BCA assay (Vazyme Biotech Co., Ltd., Nanjing, China). Proteins (25 μg) were denatured, electrophoresed, and transferred onto a 0.45 μm poly (vinylidene fluoride) (PVDF) membrane. After blocking with 5% skimmed milk for 2 h, membranes were incubated with the primary antibody overnight at 4 °C, washed, and then incubated with the secondary antibody for 2 h at room temperature. Protein bands were visualized using a Bio-Rad ChemiDoc XRS^+^ system with enhanced chemiluminescence (Beyotime Biotechnology Co., Ltd., Shanghai, China) and quantified by densitometry using Image J (1.53e).

#### 4.5.7. Histopathological Staining and Immunohistochemistry

To assess the inflammatory response, collagen deposition, and mucus secretion, lung tissues were fixed in 4% paraformaldehyde for over 24 h, followed by staining with H&E, Masson, and PAS. Histopathological changes were observed under a microscope, and inflammation was evaluated using established methods [69]. Collagen and mucus levels were quantified using Image J (1.53e) following Masson and PAS staining, respectively.

The expression levels of MMP9 and *α*-SMA in lung tissues were evaluated via immunohistochemistry. Paraffin sections (5 μm thick) were deparaffinized, rehydrated, and subjected to antigen retrieval in 0.01 M sodium citrate buffer. After blocking with diluted goat serum for 10 min, the sections were incubated with primary antibodies against MMP9 and α-SMA. Following PBS rinses, sections were incubated with horseradish peroxidase-conjugated goat anti-rabbit IgG secondary antibody for 30 min at room temperature. The sections were then developed with 3, 3′-diaminobenzidine tetrahydrochloride, counterstained with hematoxylin, and examined using a microscope system (Olympus Corporation, Tokyo, Japan). The staining intensity was quantified using Image J (1.53e).

#### 4.5.8. 16S rDNA Sequencing Analysis of Gut Microbiota

Fecal DNA was extracted using the MagPure Soil DNA LQ Kit (Guangzhou Magen Biotechnology Co., Ltd., Guangzhou, China). The V3-V4 regions of the 16S ribosomal RNA gene were amplified by PCR and sequenced on the Illumina platform (Supplementary Text S1). Raw data from the Illumina platform underwent de-multiplexing of barcoded sequences, quality filtering, noise reduction, splicing, and chimera removal to obtain representative sequences and amplicon sequence variant (ASV) abundance. Representative sequences for each ASV were selected in QIIME 2 and annotated with the Silva database (Version 138). Subsequent analyses included diversity analysis, clustering analysis, coordinates analysis, and data visualization.

#### 4.5.9. Fecal Metabolomics Analysis

Metabolites of fecal samples were analyzed using a Waters ACQUITY UPLC I-Class plus/Thermo QE HF system (Supplementary Text S2). Raw data were processed using Progenesis QI v3.0 software (Nonlinear Dynamics, Newcastle, UK). Differential metabolites were identified and analyzed using the HMDB (https://www.hmdb.ca/, accessed on 12 September 2023), Lipidmaps (https://www.lipidmaps.org/, accessed on 12 September 2023), METLIN (https://metlin.scripps.edu/, accessed on 12 September 2023), and the LuMet-animal 3.0 local database.

### 4.6. Statistical Analysis

Data are expressed as mean ± standard deviation (SD). Statistical analyses were performed using one-way analysis of variance (ANOVA) using SPSS 26.0, with a *p*-value < 0.05 considered statistically significant.

## 5. Conclusions

This study demonstrates that *Spenceria ramalana* Trimen (SRT) polyphenols exhibit significant protective effects against OVA + CS-induced asthma in rats, highlighting their potential as a multi-target therapeutic agent. SRT polyphenols effectively reduced airway inflammation by decreasing WBCs, eosinophils, and neutrophils while suppressing cytokines IL-4, IL-5, IL-13, and TNF-α. They also restored pulmonary epithelial barrier integrity by increasing ZO-1, occludin, and claudin-1 expressions. Furthermore, SRT polyphenols modulated gut microbiota composition, reducing pro-inflammatory genera such as *Prevotella* and *Parabacteroides* and enhancing beneficial taxa like *Romboutsia*, thereby emphasizing the role of the gut–lung axis. Metabolomic analyses identified altered metabolites linked to asthma-related pathways, including FcεRI signaling and galactose metabolism. Finally, SRT polyphenols downregulated the PI3K/Akt signaling pathway, alleviating airway remodeling and inflammation. These findings suggest that SRT polyphenols are a promising candidate for asthma treatment, targeting inflammation, epithelial integrity, and microbiota interactions. Future studies should explore the dose–response relationship of SRT polyphenols and validate their mechanisms using pathway-specific inhibitors. Clinical trials will be essential to confirm their efficacy and safety in humans. Investigating their potential in combination with existing asthma therapies may enhance treatment outcomes while minimizing side effects. Addressing these questions will help translate these preclinical findings into effective, nature-derived interventions for asthma and related disorders.

## Data Availability

Data will be made available upon request.

## Supplementary Materials

The following supporting information can be downloaded at: https://www.mdpi.com/article/10.3390/ijms26010165/s1.

## Abbreviations

ANOVA	analysis of variance;
α-SMA	alpha-smooth muscle actin;
BALF	bronchoalveolar lavage fluid;
BP	biological processes;
CC	cellular component;
CS	cigarette smoke;
DEX	dexamethasone;
EB	Evans blue dye;
ELISA	enzyme-linked immunosorbent assay;
Gabs	gastrointestinal absorption;
GAPDH	glyceraldehyde 3-phosphate dehydrogenase;
GO	gene ontology;
H&E	hematoxylin and eosin staining;
IL-4/5/13	Interleukin-4/5/13;
KEGG	Kyoto encyclopedia of genes and genomes;
LEfSe	linear discriminant analysis effect size;
Masson	Masson’s trichrome staining;
MF	molecular function;
MMP9	matrix metallo-peptidase 9;
MO	model;
NC	normal control;
OVA	ovalbumin;
p-Akt	phosphorylated Akt;
PAS	periodic acid–Schiff staining;
PCoA	principal co-ordinates analysis;
PI3K/Akt	phosphoinositide 3-kinase/protein kinase B;
PVDF	poly (vinylidene fluoride);
RO5	Rule of Five;
RT-qPCR	quantitative reverse transcription polymerase chain reaction;
SPF	specific-pathogen-free;
SRT	*Spenceria ramalana* Trimen;
SRTH	SRT polyphenols high-dose;
SRTL	SRT polyphenols low-dose;
Th1	type 1 T helper cells;
Th2	type 2 T helper cells;
TJs	tight junctions;
TNF-α	tumor necrosis factor-α;
UPLC-MS/MS	Ultra-Performance Liquid Chromatography-Tandem Mass Spectrometry;
W/D	wet/dry ratio of lung;
SD	standard deviation;
ZO-1	zonula occludens-1.
