# Supplementary material for: Amelioration of Inflammation in Rats with Experimentally Induced Asthma by Spenceria ramalana Trimen Polyphenols via the PI3K/Akt Signaling Pathway
Source: Int J Mol Sci. 2024 Dec 28;26(1):165. doi: 10.3390/ijms26010165 (PMC11720363; doi:10.3390/ijms26010165)
Supplement: Supplementary file 1 [file ijms-26-00165-s001.zip › ijms-3345839-supplementary.pdf]

Supplementary materials

Table S1. 84 Polyphenolic Compounds Identified in SRT

No.	Categories	Component name	Formula	Molecular weight
1	Flavonoids	Astragalin	$C_{21}H_{20}O_{11}$	448.38
2		Cosmosiin	$C_{21}H_{20}O_{10}$	432.38
3		Luteolin	$C_{15}H_{10}O_6$	286.24
4		Vitexin	$C_{21}H_{20}O_{10}$	432.38
5		Quercetin	$C_{15}H_{10}O_7$	302.24
6		Morin	$C_{15}H_{10}O_7$	302.24
7		Isoorientin	$C_{21}H_{20}O_{11}$	448.38
8		Kaempferol	$C_{15}H_{10}O_6$	286.24
9		Daidzein	$C_{15}H_{10}O_4$	254.24
10		Apigenin	$C_{15}H_{10}O_5$	270.24
11		Diosmin	$C_{28}H_{32}O_{15}$	608.54
12		Orientin	$C_{21}H_{20}O_{11}$	448.38
13		Amentoflavone	$C_{30}H_{18}O_{10}$	538.46
14		Jaceosidin	$C_{17}H_{14}O_7$	330.29
15		Afzelin	$C_{21}H_{20}O_{10}$	432.38
16		Chrysin	$C_{15}H_{10}O_4$	254.24
17		Apigenin 4'-O-rhamnoside	$C_{21}H_{20}O_9$	416.38
18	Benzoic acid and its derivatives	Ellagic acid	$C_{14}H_6O_8$	302.19
19		Gallic acid	$C_7H_6O_5$	170.12
20		4-Hydroxybenzoic acid	$C_7H_6O_3$	138.12
21		Protocatechuic acid	$C_7H_6O_4$	154.12
22		Gentisic acid	$C_7H_6O_4$	154.12
23		Vanillic acid	$C_8H_8O_4$	168.15
24		3,4-Dihydroxybenzaldehyde	$C_7H_6O_3$	138.12
25		Salicylic acid	$C_7H_6O_3$	138.12
26		2,6-Dihydroxybenzoic acid	$C_7H_6O_4$	154.12
27		Methyl gallate	$C_8H_8O_5$	184.15
28		Vanillin	$C_8H_8O_3$	152.15
29		Syringic acid	$C_9H_{10}O_5$	198.17
30		Syringaldehyde	$C_9H_{10}O_4$	182.17
31		Acetovanillone	$C_9H_{10}O_3$	166.17
32		Salicin	$C_{13}H_{18}O_7$	286.28
33		3,4-Dihydro-2H-1-benzopyran-2-one	$C_9H_8O_2$	148.16
34	Flavonols	Quercetin 3-O-glucuronide	$C_{21}H_{18}O_{13}$	478.36
35		Quercetin 3-galactoside	$C_{21}H_{20}O_{12}$	464.38
36		Isorhamnetin-3-O-glucoside	$C_{22}H_{22}O_{12}$	478.4
37		Taxifolin	$C_{15}H_{12}O_7$	304.25
38		Narcissin	$C_{28}H_{32}O_{16}$	624.54
39		Rutin	$C_{27}H_{30}O_{16}$	610.52
40		Myricetin 3-galactoside	$C_{21}H_{20}O_{13}$	480.38

41		Myricetin	$C_{15}H_{10}O_8$	318.24
42		Nicotiflorin	$C_{27}H_{30}O_{15}$	594.52
43		Isorhamnetin	$C_{16}H_{12}O_7$	316.26
44		Myricitrin	$C_{21}H_{20}O_{12}$	464.38
45	Phenylpropanoids	4-Hydroxycinnamic acid	$C_9H_8O_3$	164.16
46		1,5-Dicaffeoylquinic acid	$C_{25}H_{24}O_{12}$	516.45
47		Chlorogenic acid	$C_{16}H_{18}O_9$	354.31
48		Caffeic acid	$C_9H_8O_4$	180.16
49		Ferulic acid	$C_{10}H_{10}O_4$	194.18
50		Coniferaldehyde	$C_{10}H_{10}O_3$	178.18
51		Cryptochlorogenic acid	$C_{16}H_{18}O_9$	354.31
52		Sinapic acid	$C_{11}H_{12}O_5$	224.21
53		Sinapaldehyde	$C_{11}H_{12}O_4$	208.21
54	Flavanones	Prunin	$C_{21}H_{22}O_{10}$	434.39
55		Aromadendrin	$C_{15}H_{12}O_6$	288.25
56		Naringenin	$C_{15}H_{12}O_5$	272.25
57		Eriodictyol	$C_{15}H_{12}O_6$	288.25
58		(S)-Pinocembrin	$C_{15}H_{12}O_4$	256.25
59		Isoliquiritigenin	$C_{15}H_{12}O_4$	256.25
60		Butein	$C_{15}H_{12}O_5$	272.25
61	Coumarin and its derivatives	Daphnetin	$C_9H_6O_4$	178.14
62		Aesculin	$C_{15}H_{16}O_9$	340.28
63		Umbelliferone	$C_9H_6O_3$	162.14
64		Fraxetin	$C_{10}H_8O_5$	208.17
65		Scopoletin	$C_{10}H_8O_4$	192.17
66		Psoralen	$C_{11}H_6O_3$	186.16
67	Isoflavones	Formononetin	$C_{16}H_{12}O_4$	268.26
68		Calycosin	$C_{16}H_{12}O_5$	284.26
69		Glycitein	$C_{16}H_{12}O_5$	284.26
70		Ononin	$C_{22}H_{22}O_9$	430.4
71	Catechins and their derivatives	Catechin	$C_{15}H_{14}O_6$	290.27
72		Epicatechin	$C_{15}H_{14}O_6$	290.27
73		(±)-Gallocatechin	$C_{15}H_{14}O_7$	306.27
74		(-)-Catechin 3-O-gallate	$C_{22}H_{18}O_{10}$	442.37
75	Dihydrochalcone	Resveratrol	$C_{14}H_{12}O_3$	228.24
76		Trilobatin	$C_{21}H_{24}O_{10}$	436.41
77		Phloretin	$C_{15}H_{14}O_5$	274.27
78	Diothyrene	Phlorizin	$C_{21}H_{24}O_{10}$	436.41
79		trans-Piceid	$C_{20}H_{22}O_8$	390.38
80	Flower pigments	Delphinidin 3-glucoside	$C_{21}H_{21}ClO_{12}$	500.84
81		Pelargonidin-3-glucoside	$C_{21}H_{21}ClO_{10}$	468.84
82	Proanthocyanidins	Procyanidin B3	$C_{30}H_{26}O_{12}$	578.52
83		Procyanidin B1	$C_{30}H_{26}O_{12}$	578.52
84	Terpenoids	Perillyl alcohol	$C_{10}H_{16}O$	152.23

Table S2. Potential Active Components of SRT Polyphenols

No.	Categories	Component name	Formula	MW	Rbon	Hacc	Hdon	LogP	Gabs
1	Flavonoids	Luteolin	$C_{15}H_{10}O_6$	286.24	1	6	4	1.86	High
2		Quercetin	$C_{15}H_{10}O_7$	302.24	1	7	5	1.63	High
3		Morin	$C_{15}H_{10}O_7$	302.24	1	7	5	1.47	High
4		Kaempferol	$C_{15}H_{10}O_6$	286.24	1	6	4	1.7	High
5		Daidzein	$C_{15}H_{10}O_4$	254.24	1	4	2	1.77	High
6		Apigenin	$C_{15}H_{10}O_5$	270.24	1	5	3	1.89	High
7		jaceosidin	$C_{17}H_{14}O_7$	330.29	3	7	3	2.36	High
8		Chrysin	$C_{15}H_{10}O_4$	254.24	1	4	2	2.27	High
9	Benzoic acid and its derivatives	Ellagic acid	$C_{14}H_6O_8$	302.19	0	8	4	0.79	High
10		Gallic acid	$C_7H_6O_5$	170.12	1	5	4	0.21	High
11		4-Hydroxybenzoic acid	$C_7H_6O_3$	138.12	1	3	2	0.85	High
12		Protocatechuic acid	$C_7H_6O_4$	154.12	1	4	3	0.66	High
13		Gentisic acid	$C_7H_6O_4$	154.12	1	4	3	0.49	High
14		Vanillic acid	$C_8H_8O_4$	168.15	2	4	2	1.4	High
15		3,4-Dihydroxybenzaldehyde	$C_7H_6O_3$	138.12	1	3	2	0.79	High
16		Salicylic acid	$C_7H_6O_3$	138.12	1	3	2	1.13	High
17		2,6-Dihydroxybenzoic acid	$C_7H_6O_4$	154.12	1	4	3	0.8	High
18		Methyl gallate	$C_8H_8O_5$	184.15	2	5	3	0.97	High
19		Vanillin	$C_8H_8O_3$	152.15	2	3	1	1.57	High
20		Syringic acid	$C_9H_{10}O_5$	198.17	3	5	2	1.54	High
21		Syringaldehyde	$C_9H_{10}O_4$	182.17	3	4	1	1.66	High
22		Acetovanillone	$C_9H_{10}O_3$	166.17	2	3	1	1.77	High
23		3,4-Dihydro-2H-1-benzopyran-2-one	$C_9H_8O_2$	148.16	0	2	0	1.76	High
24	Flavonols	Taxifolin	$C_{15}H_{12}O_7$	304.25	1	7	5	1.3	High
25		Isorhamnetin	$C_{16}H_{12}O_7$	316.26	2	7	4	2.35	High
26	Phenylpropanoids	4-Hydroxycinnamic acid	$C_9H_8O_3$	164.16	2	3	2	0.95	High
27		Caffeic acid	$C_9H_8O_4$	180.16	2	4	3	0.97	High
28		Ferulic acid	$C_{10}H_{10}O_4$	194.18	3	4	2	1.62	High
29		Coniferaldehyde	$C_{10}H_{10}O_3$	178.18	3	3	1	1.75	High
30		Sinapic acid	$C_{11}H_{12}O_5$	224.21	4	5	2	1.63	High
31		Sinapaldehyde	$C_{11}H_{12}O_4$	208.21	4	4	1	1.97	High
32	Flavanones	Aromadendrin	$C_{15}H_{12}O_6$	288.25	1	6	4	1.42	High
33		Naringenin	$C_{15}H_{12}O_5$	272.25	1	5	3	1.75	High
34		Eriodictyol	$C_{15}H_{12}O_6$	288.25	1	6	4	1.62	High
35		(S)-Pinocembrin	$C_{15}H_{12}O_4$	256.25	1	4	2	2.11	High
36		Isoliquiritigenin	$C_{15}H_{12}O_4$	256.25	3	4	3	2.02	High
37		Butein	$C_{15}H_{12}O_5$	272.25	3	5	4	1.66	High
38	Coumarin and its derivatives	Daphnetin	$C_9H_6O_4$	178.14	0	4	2	1.1	High
39		Umbelliferone	$C_9H_6O_3$	162.14	0	3	1	1.44	High
40		Fraxetin	$C_{10}H_8O_5$	208.17	1	5	2	1.52	High

41		Scopoletin	$C_{10}H_8O_4$	192.17	1	4	1	1.86	High
42		Psoralen	$C_{11}H_6O_3$	186.16	0	3	0	2.01	High
43	Isoflavones	Formononetin	$C_{16}H_{12}O_4$	268.26	2	4	1	2.49	High
44		Calycosin	$C_{16}H_{12}O_5$	284.26	2	5	2	2.4	High
45		Glycitein	$C_{16}H_{12}O_5$	284.26	2	5	2	2.36	High
46		Ononin	$C_{22}H_{22}O_9$	430.4	5	9	4	2.77	High
47	Catechins and their derivatives	Catechin	$C_{15}H_{14}O_6$	290.27	1	6	5	1.47	High
48		Epicatechin	$C_{15}H_{14}O_6$	290.27	1	6	5	1.47	High
49	Dihydrochalcone	Resveratrol	$C_{14}H_{12}O_3$	228.24	2	3	3	1.71	High
50		Phloretin	$C_{15}H_{14}O_5$	274.27	4	5	4	1.41	High
51	Terpenoids	Perillyl Alcohol	$C_{10}H_{16}O$	152.23	2	1	1	2.5	High

Note: MW, molecular weight; Rbon, number of rotatable bonds; Hacc, number of H-bond acceptors; Hdon, number of H-bond donors; LogP, lipid-water partition coefficient; Gabs, Gastrointestinal absorption.

Table S3. Sequences of Primers Used for RT-qPCR

Genes		Nucleotide sequence of primers (5′ to 3′)	Length (bp)
IL-4	Forward	TCCTTACGGCAACAAGGAACA	161
	Reverse	CAGACCGCTGACACCTCTAC	
IL-5	Forward	TGACGAGCAATGAGACGATGA	132
	Reverse	ATGGTATTTCCACAGTGCCCC	
IL-13	Forward	AACCAAAAGGCCTCGGATGT	109
	Reverse	GGCCATAGCGGAAAAGTTGC	
TNF-α	Forward	ATGGGCTCCCTCTCATCAGT	106
	Reverse	GCTTGGTGGTTTGCTACGAC	
β-actin	Forward	CCCGCGAGTACAACCTTCTTG	71
	Reverse	GTCATCCATGGCGAACTGGTG	

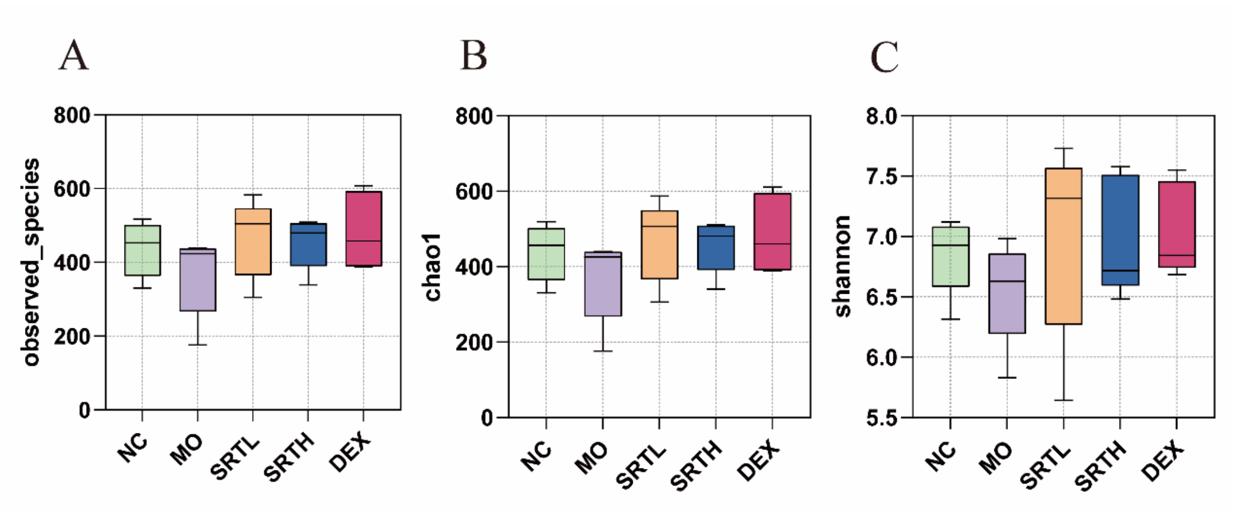

Figure S1. Gut microbial diversity. (A) Observed_species. (B) Chao1. (C) Shannon index. Data are presented as the mean ± SD (n = 5).

Text S1 Processing of Fecal Samples for 16S Sequencing

Initially, gut microbial DNA was extracted from fecal samples employing the MagPure Soil DNA LQ Kit (Guangzhou Magen Biotechnology Co., Ltd.) and quantified using agarose gel electrophoresis. Subsequently, the V3-V4 regions of the 16S ribosomal RNA gene were amplified via PCR using the forward primer 343F (5′-TACGGRAGGCAGCAG-3′) and the reverse primer 798R (5′-AGGGTATCTAATCCT-3′), with Tks Gflex™ DNA Polymerase (Takara Biomedical Technology (Beijing) Co., Ltd.). The purified PCR products were quantified using a dsDNA assay kit (Thermo Fisher Scientific Co., Ltd.). Finally, the purified amplicons were sequenced through the Illumina platform.

Text S2 Preparation of Fecal Samples and LC-MS Conditions for Metabolomics Analysis

A total of 60 mg fecal sample was added with two steel beads and 600 μL of methanol-water (V: V = 4:1, including a mixed internal standard at a concentration of 4 μg/mL) for milling at 60 Hz for 2 min, followed by ultrasonic extraction at 4°C for 10 min, and then stored at -40°C overnight. Following centrifugation for 10 min (12,000 rpm, 4°C), 200 μL of the supernatant was evaporated and reconstituted in 300 μL of methanol-water (V: V = 1: 4). Subsequently, the supernatant was evaporated to dryness, reconstituted in 300 μL of methanol-water (V: V = 1:4), and allowed to remain at -40°C for 2 h. Ultimately, the samples were centrifuged for 10 min (12,000 rpm, 4 °C), and the supernatant was filtered through a 0.22 μm filter in preparation for LC-MS analysis. Quality control (QC) samples were prepared as a mixture of equal volumes of all samples and subjected to four consecutive scans to evaluate stability and reliability.

The LC-MS analysis platform consisted of an ACQUITY UPLC I-Class plus system (Waters Corp., Milford, MA, USA) and a Q Exactive HF mass spectrometry detector (Thermo Fisher Scientific Co., Ltd., Massachusetts, USA) equipped with both positive and negative ion source electrospray capabilities. 2 μL samples were injected and separated by an ACQUITY UPLC HSS T3 (100 mm × 2.1 mm, 1.8 μm) at 45°C, with the mobile phase maintained at a flow rate of 0.35 mL/min. The optimized mobile phase and gradient elution conditions were as follows: mobile phase A was an aqueous solution containing 0.1% formic acid, while mobile phase B comprised acetonitrile. The gradient elution procedure was as follows: 0-2 min, 95% A; 2-4 min, 95-70% A; 4-8 min, 70-50% A; 8-10 min, 50-20 % A; 10-14min, 20-0% A. To maximize the detection of high fragment ions, a wide m/z range of 100–1200 was scanned employing both positive and negative ion modes. The electrospray ion source conditions were as follows: spray voltage 3.8 kV, capillary temperature 320°C, aux gas heater temperature 350°C, sheath gas flow rate 35 Arb, and aux gas flow rate 8 Arb. A mixed internal standard (2-chloro-L-phenylalanine, succinic acid-d4, L-valine-d8, cholic acid-d4) was used as a lock-mass solution for accurate mass calibration to enhance accuracy and consistency.
